# Real-Life Treatment Paradigms Show Adalimumab Is Cost-Effective for the Management of Ulcerative Colitis

**DOI:** 10.1155/2016/5315798

**Published:** 2016-10-03

**Authors:** Candace L. Beilman, Nguyen Xuan Thanh, Victoria Ung, Christopher Ma, Karen Wong, Karen I. Kroeker, Thomas Lee, Haili Wang, Arto Ohinmaa, Phil Jacobs, Brendan P. Halloran, Richard N. Fedorak

**Affiliations:** ^1^Division of Gastroenterology, University of Alberta, Edmonton, AB, Canada; ^2^Institute of Health Economics, Edmonton, AB, Canada

## Abstract

*Background*. Adalimumab is effective for the maintenance of remission in patients with moderate-to-severe ulcerative colitis (UC). Currently, biologic therapies are used in cases where patients fail conventional medical therapies. If biologic therapies are not available, patients often choose to remain in an unwell state rather than undergo colectomy.* Objective*. The aim of the study was to evaluate the cost-effectiveness of adalimumab in patients with UC where adalimumab was readily available compared to not available.* Methods*. A previously validated Markov model was used to simulate disease progression of patients with UC who are corticosteroid-dependent and/or did not respond to thiopurine therapy. Utility scores and transition probabilities between health states were determined by using data from randomized controlled trials and real-life observational studies. Costs were obtained from the Ontario Case Costing Initiative and the Alberta Health Schedule of Medical Benefits.* Results*. The incremental cost-effectiveness ratios for readily available adalimumab treatment of UC were $40,000 and $59,000 per quality-adjusted life year, compared with ongoing medical therapy in an unwell state, at 5-year and 10-year treatment time horizons, respectively.* Conclusion*. Considering real-life patient preferences to avoid colectomy, adalimumab is cost-effective according to a willingness-to-pay threshold of $80,000 for treatment of UC.

## 1. Introduction

Ulcerative colitis (UC) is a chronic, relapsing, and remitting inflammatory disease that is characterized by symptoms of diarrhea, rectal bleeding, urgency, and abdominal pain [1]. Canada has one of the highest incidence rates of UC in the world and this incurs a considerable burden to the Canadian healthcare system, with direct and indirect costs close to $1 billion per year [2].

Due to the relapsing-remitting course of UC, treatment is focused on the induction and maintenance of clinical remission and endoscopic mucosal healing [1]. Maintaining remission requires continuous medical therapy and ongoing monitoring of disease activity. Conventional medical therapies, including mesalamine, corticosteroids, and oral immunosuppressants (azathioprine, 6-mercaptopurine), may be inadequate to maintain clinical remission in some patients [3].

For patients who fail to maintain remission on the above therapy, the options for treatment are limited to continuous corticosteroid use, colectomy, or biologic therapy. The chronic use of corticosteroids is associated with significant adverse effects and can leave the patient in a chronically unwell state [4]. While colectomy with a permanent ileostomy or an ileoanal pouch procedure can result in improved quality of life, it may be associated with significant morbidity and can lead to concerns with body image [5, 6]. In this regard, patients often delay the colectomy and elect to remain in a chronically unwell state, often on chronic corticosteroids.

The use of the antitumor necrosis factor alpha (TNF*α*), adalimumab, has been shown to be well tolerated and effective in inducing remission in patients with moderate-to-moderately severe active UC [3, 7–10]. The benefits of adalimumab for patients with inflammatory bowel disease (IBD) include increased quality of life, steroid discontinuation, and reduced hospitalization and surgery rates [9, 11].

The cost of biologic therapy is significant, varying from $18,000 to $33,000 per patient per year [12]. While there is limited cost-effective data of adalimumab for the treatment of UC, there are several conflicting studies examining its cost in Crohn's disease. One study found adalimumab to be cost-effective compared to conventional therapies for maintenance and remission in patients with active Crohn's disease, with an ICER of *₤*17 873 per quality-adjusted life year (QALY) over a 1-year time period [13]. Similarly, another study, using a lifetime Markov Model concluded that adalimumab was more economical compared to standard care of Crohn's disease, with an ICER of *₤*7190/QALY for 1 year of treatment [14]. In contrast, Blackhouse et al. suggested that adalimumab was not cost-effective in managing patients with Crohn's disease compared to usual care, with an ICER of $US193,305/QALY over a 5-year period [15]. The conflicting nature of these results suggests discrepancies in methods and definitions used, costs included, and countries where the study took place. While economic data exists for adalimumab treatment of Crohn's disease, there is currently no cost-effectiveness study for adalimumab treatment in ulcerative colitis.

In this analysis, the costs and utility of patients receiving readily available adalimumab treatment for ulcerative colitis was compared to that where adalimumab was not readily available and thus the patient preference for a chronically unwell state, with or without corticosteroids, rather than immediate colectomy dominated [16–18].

## 2. Methods

### 2.1. Type of Study and Outcome

Our study replicated a previously validated Markov model in ulcerative colitis that was conducted by our center for another anti-TNF agent, infliximab, that calculates the difference in costs divided by the differences in utility between the study option and alternative intervention, the result being the incremental cost-effectiveness ratio (ICER) [19]. In the current study, this validated the fact that anti-TNF Markov model was replicated for the use of adalimumab treatment of UC.

### 2.2. Target Population

A base-case analysis was used that consisted of a theoretical cohort of patients with moderate-to-severe active UC who are corticosteroid-dependent and either failed or are intolerant to thiopurine treatment. Approximately 60% were male, with an average age of 40 years old.

### 2.3. Markov Model

**Figure 1:**
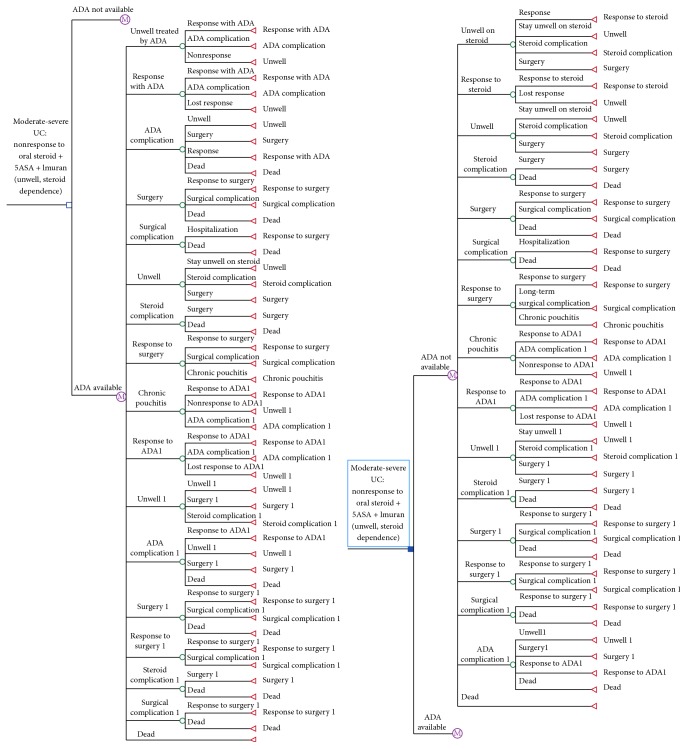
Markov model simulating the progression of a cohort of patients with moderate-to-severe ulcerative colitis, who are corticosteroid-dependent or refractory to thiopurines, in situations where adalimumab is readily available compared to situations when it is unavailable.

A previous established Markov model was used to determine the ICER of two management strategies: (1) no adalimumab, which includes scenarios where adalimumab was not available and patients therefore remained in a chronically unwell state in order to avoid colectomy, and (2) adalimumab therapy, where adalimumab was readily available to induce and maintain clinical response. Patients in this group were modeled as being treated initially with 160 mg, 80 mg at week 2, followed by 40 mg every other week. The Markov model structure is displayed in Figure 1.

The different health states used in the model were defined and verified by a panel of gastroenterologists and gastrointestinal surgeons with expertise and experience in the treatment of inflammatory bowel disease (Table 2). Based on their management strategy, patients were assigned to an initial health state of 3 months and were evaluated every 3 months over a 5-year (20 cycles) time horizon. At the end of every 3-month cycle, patients were assigned probabilities of moving on to ensuing health states.

The probabilities of moving on to subsequent health states were as follows: patients who received adalimumab therapy either experienced an induction response or became “nonresponders.” The patients who responded to adalimumab may continue to respond to treatment over time or they may experience a secondary loss of response. The patients who did not respond to the initial adalimumab treatment or who lost response to treatment returned to ongoing steroid therapy, where a portion of patients eventually underwent colectomy. Patients who experienced an adverse effect due to adalimumab therapy could sometimes be successfully treated for the complication. If they could not be treated for the complication, they were either taken off adalimumab and returned to ongoing steroid therapy or offered a colectomy. Patients who received a colectomy could develop complications associated with the surgery or could remain in a response state. If a patient develops chronic pouchitis due to surgery, they can be subsequently treated with adalimumab or return to steroid treatment. The possibility of patient mortality was considered for each health state.

### 2.4. Model Inputs

Our analysis follows the 2006 economic evaluation guidelines as set out by the Canadian Agency for Drugs and Health Technologies [20].

#### 2.4.1. Transition Probabilities

The probabilities of patients moving between health states were derived from a review of the literature from both randomized controlled trials and real-life studies. Study results were weighted based on sample size. Loss of response to adalimumab was obtained from our center data in an attempt to replicate real-life clinical response. The weighted probabilities were then reviewed by the panel of gastroenterologists for face validity. Tables 3 and 4 show the transition probabilities associated with each health state.

#### 2.4.2. Costs of Health States

A literature search was administered to assess the costs of each health state per 3-month cycle. To estimate resource use, we included physician, hospital, and outpatient drug costs. Physicians' fees were obtained from the Alberta provincial fee schedules of Alberta Health and Wellness [21]. Hospital costs for all hospitalization episodes came from the Ontario Case Costing Initiative [22]. The costs of drugs were obtained from the Alberta Health and Wellness Drug Benefit List [12]. The costs of corticosteroid, adalimumab, or surgical-complication health states were estimated by averaging the cost of complication weighted by the likelihood of occurrence. Cost of death was counted once and equal to the cost of the health state that led to the death. Tables 3 and 4 outline the costs of each health state per 3-month cycle.

#### 2.4.3. Utility

In order to calculate quality-adjusted life years (QALYs) for each treatment regimen, utilities for each health state were determined through a review of the literature and access to expert opinion. The utility value assigned to each health state is outlined in Tables 3 and 4.

### 2.5. Sensitivity Analysis

One-way sensitivity analyses with tornado diagrams (available on request) were conducted on all key parameters for 6 scenarios with a time horizon of 5, 10, or 15 years, and with a utility score of the response-to-adalimumab health state as 0.79 or 0.82. The probabilities and utility scores were varied between the lower and upper ends of 95% confidence interval, and the cost of each health state was varied by 25%, as shown in Tables 3 and 4. Also, a probabilistic sensitivity analysis for costs and utility scores was performed. A normal distribution was used for costs and a beta distribution for utility scores that are far from 0; utility scores close to 0 were transformed to utility decrement (= 1 − *u*), and a gamma distribution was used.

### 2.6. Probabilities of Response

As shown in Table 5, patients on adalimumab tend to lose response over time [23, 24], and loss of response generally requires additional interventions such as dose escalation, rescue steroids, or surgical intervention. Loss of response rates were used to demonstrate the effectiveness of adalimumab, with nonresponse defined using clinical disease activity indices, inflammatory markers, and endoscopic and radiographic evidence of disease activity. Adalimumab levels were not routinely available at our center during the study inclusion period and were therefore not used in our analysis. The average loss of response rates to adalimumab for each 3-month cycle up to 20 cycles (Table 5) were collected from data obtained by Ma et al. [23] at the University of Alberta Inflammatory Bowel Disease Consultation and Research Clinic, Edmonton, Alberta, Canada. It was assumed that, after 20 cycles, loss of response rates plateaued.

In a retrospective cohort study from our expert IBD center, it was determined that dose escalation was required in 50% of UC patients after a mean time span of 59.3 (±70.5) weeks [23]. Dose escalation of adalimumab typically consists of increasing the dose to 80 mg or increasing to weekly injections of 40 mg. Currently, there is a lack of research that examines the response rates of dose escalation in UC patients. Thus, it was agreed by collaboration with a gastroenterology expert panel that loss of response rates in UC after dose escalation would be fixed to that seen for Crohn's disease; therefore our expert IBD center Crohn's disease outpatient data was used to estimate loss of response to dose escalation at each 3-month cycle (Table 5).

The significant amount of patients needing dose escalation and the high costs of dose escalation results in many centers attempting dose de-escalation. To determine the cost-effectiveness of dose de-escalation, we assumed that dose de-escalation was attempted in 54% of patients and was successful in 63% of those patients, based on data obtained by Baert et al. [25]. When determining the utility score for a response to adalimumab, we obtained 2 different score values that represent the utility of patients in remission. Using 2 different estimation methods, the utility scores for patients with steroid-refractory ulcerative colitis were 0.79 by time trade-off and 0.82 by visual rating scale [26].

### 2.7. Discounting

Costs and utility scores were discounted annually at the rate of 5%.

## 3. Results

### 3.1. Costs and Quality-Adjusted Life Years

#### 3.1.1. Adalimumab Is Not Available and Patients Opt for an Ongoing Unwell State

With a utility score of 0.79, the cost-utility analysis yielded a cost of $97,000 with 3.154 QALYs for a patient during a 10-year period.

#### 3.1.2. Adalimumab Is Available and Patients Opt for Adalimumab Treatment of Induction and Maintenance

When adalimumab is readily available and patients opt for adalimumab treatment to induce and maintain response, the cost-utility analysis with a utility score of 0.79 yielded a cost of $107,000 with 3.321 QALYs for a patient during a 10-year period.

### 3.2. Incremental Cost-Effectiveness Ratio

The ICER at 10 years, when comparing readily available adalimumab treatment to ongoing medical therapy in an unwell state, was $59,000 per QALY gained when using a utility score of 0.79 measured by time trade-off and $53,000 per QALY gained when using a utility score of 0.82 measured by visual rating scale (Table 1).

Sensitivity analysis of probabilities, costs, and utility scores showed that the ICER varied from $37,000 to $81,000 (utility score 0.79) or from $33,000 to $72,000 (utility score 0.82) at the 10-year horizon (Table 1). The most sensitive variables were the cost of response to adalimumab and the utility of an unwell state, whereas the least sensitive variables were the probability of surgical complication and the probability of hospitalization among surgical complications.

Sensitivity analyses were also performed with varying time horizons. The ICER of adalimumab therapy versus no adalimumab therapy ranged from $25,000 to $65,000 (utility score of 0.79) and from $22,000 to $58,000 (utility score of 0.82) at a 5-year horizon. At a 15-year horizon, the ICER ranged from $45,000 to $91,000 (utility score of 0.79) and from $40,000 to $81,000 (utility score of 0.82).

### 3.3. Incremental Cost-Effectiveness Ratio Associated with Adalimumab Dose Escalation

Upon analyzing dose escalation response rates using Crohn's disease data, we estimated dose escalation ICERs to be $85,000 at 5 years, $102,000 at 10 years, and $113,000 at 15 years when using a utility score of 0.79. Analysis of dose escalation with a utility score of 0.82 revealed ICERs of $77,000 at 5 years, $92,000 at 10 years, and $102,000 at 15 years.

### 3.4. Incremental Cost-Effectiveness Ratio Associated with Adalimumab Dose De-Escalation

The ICERs associated with dose de-escalation were $75,000 at 5 years, $93,000 at 10 years, and $105,000 at 15 years when applying a utility score of 0.79 to the response-to-adalimumab health state. Analysis of dose de-escalation with a utility score of 0.82 revealed ICERs of $63,000, $84,000, and $95,000, at 5, 10, and 15 years, respectively.

### 3.5. Cost-Effectiveness Acceptability

**Figure 2:**
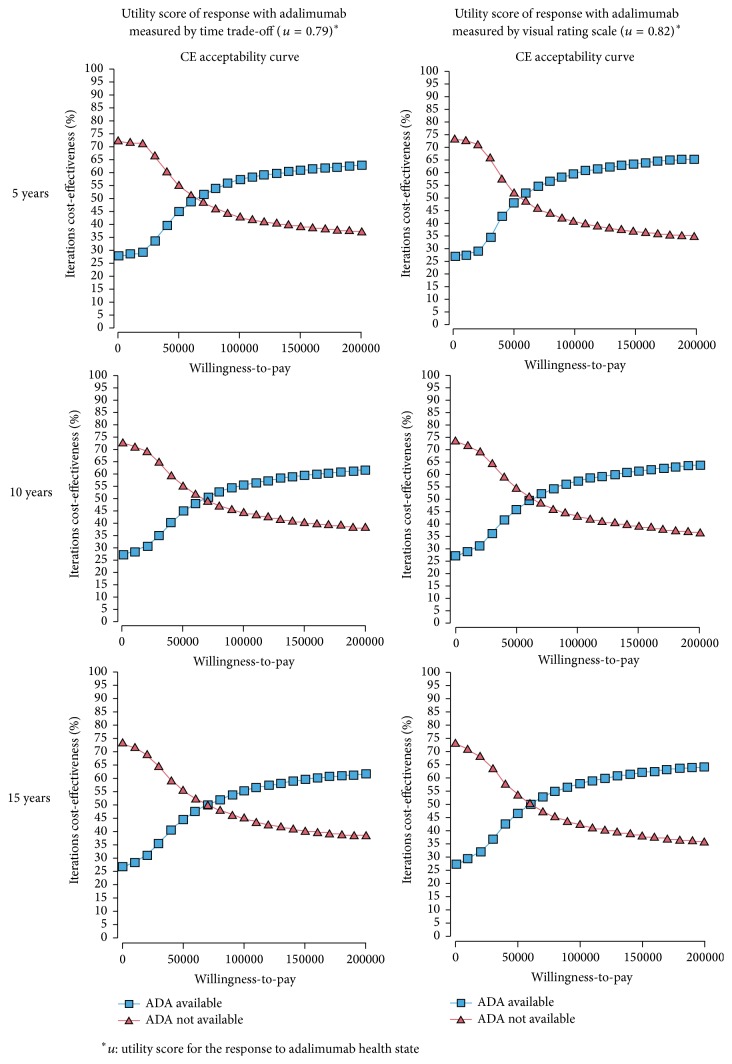
Cost-effectiveness acceptability curves.

Cost-effectiveness acceptability curves are presented in Figure 2. Given a time horizon of 10 years and a utility score of 0.79 for a response to adalimumab, the graph shows a 45% chance that adalimumab treatment will be cost-effective if the willingness-to-pay for an extra QALY is $50,000. The probability of adalimumab treatment being cost-effective if the willingness-to-pay is $100,000 and $150,000 is 56% and 60%, respectively. Using the same time horizon (10 years) with a utility score of a response to adalimumab health state to be equal to 0.82, the probability of adalimumab treatment being cost-effective is 46%, 57%, and 61% at a willingness-to-pay of $50,000, $100,000, and $150,000, respectively.

## 4. Discussion

Currently, two multicenter, randomized, placebo-controlled trials have shown adalimumab to be well tolerated and effective in inducing and maintaining remission in patients with moderate-to-severe UC [3, 9], in addition to an abundance of open-label studies that further support these findings [7, 8, 23, 24, 27–29]. The emergence of adalimumab and other biologic agents has given UC patients an additional treatment option to consider once they have become corticosteroid-dependent. While considering any treatment strategy, the costs of therapy must be taken into consideration regardless of outcome. In order to determine if a strategy is worthwhile, a willingness-to-pay threshold must be set.

To date, there is no willingness-to-pay threshold that is accepted universally throughout healthcare systems. A threshold value of $50,000 per QALY gained has been widely used in many studies and countries as a reference threshold since the 1970s, although its use is often debated as being too low. Grosse argued that the $50,000 per QALY threshold is an arbitrary decision rule that lacks theoretical and empirical justification and is outdated due to the failure to adjust the value for inflation or changing levels of income or healthcare budgets since its introduction [30]. Also, it should be noted that different medical conditions have different willingness-to-pay thresholds, depending on the severity of the disease [31]. The Canadian Drug Expert Committee between 2003 and 2007 has accepted therapies up to $80,000, further demonstrating the ambiguity in the determination of an appropriate willingness-to-pay threshold [32]. Due to the long-lasting and debilitating nature of UC, we assume that a threshold of $80,000 is appropriate to consider the cost-effectiveness of adalimumab in the treatment of UC.

Based on the $50,000 willingness-to-pay threshold, it appears that adalimumab therapy is cost-effective compared to ongoing less effective medical therapies at a 5-year time horizon. Although the ICERs for 10-year and 15-year time horizons surpass that threshold, they are all considered to be cost-effective according to a willingness-to-pay threshold of $80,000 per QALY gained as per Rocchi et al. [32]. These results demonstrate that although the cost of adalimumab is significant, it presents as a worthwhile treatment option in patients with moderate-to-severe active UC.

By using the previously validated Markov model validated for infliximab [19] as the model for this current study, we are able to compare the cost-effectiveness of the two main biologic agents currently in use for the management of UC: infliximab and adalimumab. Our original infliximab analysis demonstrated infliximab to have an ICER of $US64,000 and $US79,000 at 5 and 10 years, respectively, therefore being cost-effective at a willingness-to-pay threshold of $US80,000. The current study demonstrated adalimumab to have an ICER of $US44,000 and $US58,000 at 5 and 10 years, respectively, when converted to $US using the conversion rate implemented in the infliximab study. Given these results, it appears that adalimumab, at the cost available in Canada ($740.36/40 mg), may be similar or numerically more cost-effective for the management of moderate to severe active UC than infliximab. However, we recommend that further economic analyses should compare these two anti-TNF agents directly. Also, the introduction of new biologic therapies and biosimilars for ulcerative colitis results in a need to assess how the cost profile of these agents could potentially be affected.

The lower ICER for adalimumab compared to infliximab is likely due to the lower cost of adalimumab in Canada compared to infliximab per 3-month cycle. In addition, this difference may be larger than expected due to the lack of the infliximab model taking into consideration the indirect costs of infliximab administration. Infliximab administration requires patients to receive intravenous injections at an outpatient health center, opposed to adalimumab which can be administered by the patients subcutaneously. Examining the cost-effectiveness of adalimumab and infliximab in separate models is a limitation of our study, as in real-life practice, patients are able to switch between these two agents.

An exploratory analysis of ICERs associated with dose escalation was conducted due to the high rate of patients who undergo dose escalation as a result of secondary loss of response. The average ICER associated with dose escalation was $85,000, $102,000, and $113,000 at 5, 10, and 15 years, respectively. This data clearly demonstrates that a need for dose escalation in patients who experience a loss of response to adalimumab results in additional costs, thus increasing the ICER above frequently used willingness-to-pay thresholds. Furthermore, dose de-escalation is common in patients who regain response after being dose escalated. Our analysis revealed ICERS of $75,000 at 5 years, $93,000 at 10 years, and $105,000 at 15 years, indicating that dose de-escalation has the potential to reduce costs after patients have regained response to adalimumab upon dose escalation.

An important limitation of our study is the lack of our model taking into consideration the use of concomitant therapies, including immunosuppressants and methotrexate. Numerous studies have demonstrated a difference in the efficacy of anti-TNF agents and steroids with the use of combination therapy [33, 34]. For our model, we assumed that the use of combination therapy was equivalent in both the adalimumab and chronic steroid treatment arm.

In conclusion, this study demonstrates that, using response rates from real-life centers and real-life patient preference to avoid colectomy, readily available adalimumab treatment of ulcerative colitis is cost-effective according to willingness-to-pay thresholds of $80,000 per QALY compared with when adalimumab is not available and the patients elect for a chronic unwell state to avoid colectomy. Dose escalation will increase these costs.

## Figures and Tables

**Table 1:** Incremental cost-effectiveness ratios between adalimumab treatment and no adalimumab treatment but instead ongoing medical treatment.

Time horizon	Utility score of response with ADA measured by time trade-off (*u* = 0.79)^*∗*^	Utility score of response with ADA measured by visual rating scale (*u* = 0.82)^*∗*^
5 years	$45,000 ($25,000–$65,000)	$40,000 ($22,000–$58,000)
10 years	$59,000 ($37,000–$81,000)	$53,000 ($33,000–$72,000)
15 years	$68,000 ($45,000–$91,000)	$60,000 ($40,000–$81,000)

^*∗*^ *u* = utility score for the response to adalimumab health state.

**Table 2:** Health state definitions.

Health state	Definition
Response to medical treatment (steroid/ADA)	Reduction/resolution of symptoms due to patients' respective treatment regimens. Patients in this cohort would have a UC Disease Activity Index (DAI) score of 0–2 (out of 12) or a partial Mayo score of 0-1 (out of 9).

Unwell	Patients are experiencing recurrent disease activity despite being treated with medical therapy (steroids, 5-ASA, azathioprine, or biologics). Patients in this cohort would have a UC DAI score of 3–8 (out of 12) or a partial Mayo score of 2–6 (out of 9). Symptoms often include 5–8 bowel movements per day, some rectal bleeding, and chronic fecal urgency.

Chronic pouchitis	A common long-term complication after restorative proctocolectomy with ileal pouch-anal anastomosis for patients with UC. Chronic pouchitis is characterized by inflammation of the ileal pouch after surgery, presenting with symptoms of increased stool frequency, urgency, incontinence, and dehydration. In this study, the chronic pouchitis health state refers to chronic pouchitis patients who are refractory to antibiotic therapy to attain remission.

Steroid/ADA complication	Any complication that occurred as a result of the medical treatment (steroid or ADA) that required a change in treatment or health state.

Non-/loss of response (ADA)	Nonresponse refers to patients who never responded to ADA, whereas loss of response refers to patients who experienced an initial response but lost response in subsequent cycles.

Surgery	Proctocolectomy with ileal pouch-anal anastomosis for those patients who did not respond to medical treatment. Typically patients with severe UC undergo surgery in order to manage their disease.

Surgical complication	Any complication that occurred as a result of surgery that requires patient to be hospitalized or to undergo further surgery to correct the complication.

**Table 3:** Markov model input parameters for chronic ulcerative colitis.

	Transition probabilities (%)/cycle	Costs (CA$)/cycle	Utility scores/year
*(A) Ongoing steroids* [35, 36]		**917 (±25%)**	**0.32 (±0.31)**
(1) Response	33.92 (28.09–40.33)		
(2) Unwell	57.11 (50.5–63.27)		
(3) Complication	2.80 (0.56–7.63)		
(4) Surgery	#		

*(B) Response to steroids* [35]		**0**	**0.79 (±0.21)**
(1) Response	53.30 (46.81–59.67)		
(2) Loss of response	#		

*(C) Unwell on steroids* [35, 36]		**917 (±25%)**	**0.32 (±0.31)**
(1) Unwell	#		
(2) Complication	2.80 (0.56–7.63)		
(3) Surgery	10.00 (6.40–14.28)		

*(D) Steroid complication* [35]		**23,919 (±25%)**	**0.16 (±0.16)**
(1) Surgery	98.00 (93.70–99.78)		
(2) Death	#		

*(E) Surgery* [37, 38]		**37,159 (±25%)**	**0.16 (±0.16)**
(1) Early response	#		
(2) Complication	12.8 (8.76–17.91)		
(3) Death	2.50 (0.98–5.69)		

*(F) Response to surgery* [37, 39]		**0**	**0.58 (±0.15)**
(1) Response to surgery	85.80		
(2) Surgical complication	#		
(3) Chronic pouchitis (CP)	11.70		

*(G) Chronic pouchitis* [40, 41]		**8,144 (±25%)**	**0.32 (±0.31)**
(1) Response to ADA (CP)	See Table 5		
(2) Nonresponse (unwell-CP)	#		
(3) ADA complication (CP)	4.20		

*(H) Surgical complication* [38]		**17,586 (±25%)**	**0.49 (±0.32)**
(1) Hospitalization	99.50 (97.22–99.99)		
(2) Death	#		

*(I) Adalimumab (ADA)* [23]		**8,144 (±25%)**	**0.32 (±0.31)**
(1) Response to ADA	86.80 (75.74–97.86)		
(2) ADA complication	3.04		
(3) Nonresponse (unwell)	#		

*(J) Response to ADA*		**4,442 (±25%)**	**0.79–0.82**
(1) Response to ADA	See Table 5		
(2) ADA complication	7.88		
(3) Loss of response (unwell)	#		

*(K) Adalimumab complications*		**12,059 (±25%)**	**0.16 (±0.16)**
(1) Response to ADA	70.00		
(2) Unwell on steroids	14.00		
(3) Surgery	14.00		
(4) Death	#		

*(L) Death*	1	*Equal to cost of corresponding health state*	**0**

#: complement probability.

**Table 4:** Markov model input parameters for chronic pouchitis.

Health states	Transition probabilities (%)/cycle	Costs (CA$)/cycle	Utility scores/year
*(M) Response to ADA 1*		**4,442 (±25%)**	**0.58 (±0.15)**
(1) Response to ADA 1	See Table 5		
(2) Lost response (unwell 1)	#		
(3) ADA complication 1	4.20		

*(N) Unwell 1*		**917 (±25%)**	**0.32 (±0.31)**
(1) Unwell 1	#		
(2) Surgery 1	10.00		
(3) Steroid complication 1	2.80		

*(O1) ADA complication 1 in the ADA not available arm*		**12,059 (±25%)**	**0.16 (±0.16)**
(1) Response to ADA 1	60.00		
(2) Unwell 1	19.00		
(3) Surgery 1	19.00		
(4) Death	#		

*(O2) ADA complication 1 in the ADA available arm*		**12,059 (±25%)**	**0.16 (±0.16)**
(1) Response to ADA 1	51.00		
(2) Unwell 1	23.50		
(3) Surgery 1	23.50		
(4) Death	#		

*(P) Surgery 1 (permanent ileostomy)*		**37,159 (±25%)**	**0.16 (±0.16)**
(1) Response 1	#		
(2) Surgery complication 1	12.80		
(3) Death	2.50		

*(Q) Steroid complication 1*		**23,919 (±25%)**	**0.16 (±0.16)**
(1) Surgery 1	98.00		
(2) Death	#		

*(R) Response to surgery 1*		**0**	**0.44 (±0.11)**
(1) Response to surgery 1	#		
(2) Surgical complication 1	2.50		

*(S) Surgical complication 1*		**17,586 (±25%)**	**0.37 (±0.24)**
(1) Response to surgery	#		
(2) Death	0.50		

#: complement probability.

**Table 5:** Maintenance probabilities of patients on adalimumab over time.

Cycle #	Rate of response of UC patients [23]	Chronic pouchitis patients (ADA not available arm) [40, 41]	Chronic pouchitis patients (ADA available arm)^*∗*^	Rate of response of patient's dose escalated [42] ^*∗∗*^
0	86.8	62.6	53.2	93.8
1	73.3	61.2	52.0	80.5
2	66.5	59.9	50.9	73.7
3	62.0	58.6	49.8	69.1
4	58.7	57.3	48.7	65.8
5	56.2	56.1	47.7	63.2
6	54.1	54.9	46.7	61.1
7	52.4	53.7	45.6	59.4
8	50.9	52.5	44.6	57.8
9	49.6	51.4	43.7	56.5
10	48.5	50.3	42.8	55.3
11	47.5	49.2	41.8	54.3
12	46.5	48.1	40.9	53.3
13	45.7	47.0	40.0	52.5
14	45.0	46.0	39.1	51.7
15	44.3	45.0	38.3	51.0
16	43.6	44.0	37.4	50.3
17	43.0	43.1	36.6	49.7
18	42.4	42.1	35.8	49.1
19	41.9	41.2	35.0	48.5
≥20	41.4	40.2	34.2	48.0

^*∗*^To calculate the response probability for patients with chronic pouchitis who had been previously exposed to and failed adalimumab, a 15% discount was taken from the probability of response of patients with chronic pouchitis who had never been exposed to ADA.

^*∗∗*^These maintenance probabilities are based on Crohn's disease patient information.
